# Validation of the Hungarian version of the CarerQol instrument in informal caregivers: results from a cross-sectional survey among the general population in Hungary

**DOI:** 10.1007/s11136-020-02662-8

**Published:** 2020-10-10

**Authors:** Petra Baji, Werner B. F. Brouwer, Job van Exel, Dominik Golicki, Valentina Prevolnik Rupel, Zsombor Zrubka, László Gulácsi, Valentin Brodszky, Fanni Rencz, Márta Péntek

**Affiliations:** 1 Department of Health Economics, Corvinus University of Budapest, Fővám tér 8, Budapest, 1093 Hungary; 2 Erasmus School of Health Policy & Management (ESHPM), Erasmus University Rotterdam, PO Box 1738, 3000 DR Rotterdam, The Netherlands; 3 Erasmus School of Economics (ESE), Erasmus University Rotterdam, PO Box 1738, 3000 DR Rotterdam, The Netherlands; 4 Department of Experimental and Clinical Pharmacology, Medical University of Warsaw, ul. Banacha 1b, 02-097 Warsaw, Poland; 5 Institute for Economic Research, Kardeljeva ploščad 17, 1000 Ljubljana, Slovenia; 6 Health Economics Research Center, University Research and Innovation Center, Óbuda University, Bécsi út 96/B, Budapest, 1034 Hungary; 7 Premium Postdoctoral Research Programme, Hungarian Academy of Sciences, Nádor u. 7, Budapest, 1051 Hungary

**Keywords:** Informal care, Burden, Validation, CarerQol, EQ-5D-5L

## Abstract

**Purpose:**

The CarerQol instrument has been designed and validated as an instrument able to measure both the positive and the negative impacts of caregiving on the quality of life of informal caregivers (CarerQol-7D), as well as their general happiness (CarerQol-VAS). The aim of this study was to assess the construct validity of the CarerQol in the Hungarian context.

**Methods:**

The CarerQol was translated into Hungarian. Subsequently, in a cross-sectional online survey, representative for the general Hungarian population (*N* = 1000), informal caregivers were identified (*N* = 149, female 51.2%, mean age 53.2). Clinical, convergent and discriminant validity of the CarerQol were evaluated in relation to the caregivers’ and care recipients’ EQ-5D-5L health status, and caregiving situation characteristics.

**Results:**

Average CarerQol-7D and CarerQol-VAS scores were 76.0 (SD 16.2) and 6.8 (SD 2.3), respectively. CarerQol-7D and CarerQol-VAS scores were significantly correlated with caregiving time (*r* = − 0.257; − 0.212), caregivers’ EQ-5D-5L scores (*r* = 0.453; 0.326) and the CarerQol-7D also with care recipients’ EQ-5D-5L scores (*r* = 0.247). CarerQol-7D scores differed significantly with relevant caregiving characteristics (e.g. nature and severity of care recipients’ health status, sharing household) and both the CarerQol-7D and CarerQol-VAS with the overall care experience.

**Conclusion:**

Our findings confirmed the validity of the Hungarian language version of the CarerQol and support the cross-cultural validity of the instrument. CarerQol-7D scores performed better in distinguishing caregiving situation characteristics than the general happiness measure CarerQol-VAS. Care recipients’ health status was only weakly associated with informal caregivers’ care-related quality of life and happiness. Caregivers’ own health and caregiving circumstances were more strongly associated with these scores.

**Electronic supplementary material:**

The online version of this article (10.1007/s11136-020-02662-8) contains supplementary material, which is available to authorized users.

## Introduction

Care provided by family members, friends or other acquaintances, often unpaid, makes up a significant part of the total amount of long-term care provided to individuals in need of help or support due to health problems or frailty as a consequence of ageing. This type of voluntary help is commonly called informal care. Estimates suggest that the prevalence rates of informal care vary from 20 up to 44% in Europe [1]. Informal care has an important impact in economic, social and health terms, particularly in the context of chronic diseases. Especially in countries with tight financial and capacity constraints in their health and social care sectors, and which face ageing populations, informal care is an indispensable part of the total care provided to people in need [2, 3]. It has therefore been advocated to include the effects of interventions on informal caregivers in policy decision-making and (economic) evaluations of health and social care interventions [4, 5]. Such inclusion can have a significant impact on cost-effectiveness of health and social care interventions [6]. Hence, there is an increasing need to measure and value informal care, using validated methods that allow comparisons across populations with different caregiving contexts, help to reduce the methodological heterogeneity and increase transferability of results across countries.

Providing informal care can strongly influence the quality of life of the caregiver as the tasks involved can be physically and emotionally demanding, especially when required over longer periods of time and with high intensity [7, 8]. On the other hand, studies confirmed that caring for a family member or friend can be fulfilling as well [9, 10]. For example, Brouwer et al. [10] showed that, given the illness of a loved one and their need for informal care, the happiness of most caregivers would decrease if they had to hand-over all care tasks to someone else. Hence, informal caregiving can impact several life domains of caregivers, either negatively or positively. The CarerQol questionnaire has been developed to quantify and value the subjective burden of providing informal care, by measuring care-related quality of life of informal caregivers [7, 11, 12]. The CarerQol comprises a descriptive system with seven items (CarerQol-7D) covering both positive and negative impacts of informal care and a visual analogue scale measuring happiness (CarerQol-VAS).

Translated versions of the CarerQol have been developed for several languages already (i.e. English, Dutch, German, Norwegian, Swedish, Italian, Spanish and Portuguese) and these have been successfully used in multi-country surveys [13, 14]. However, language versions for the Central and Eastern European region are currently still lacking. Moreover, notwithstanding exceptions like a study in the USA [15] and one in Australia [16], studies focusing on the validity of the instrument were performed in The Netherlands. Given the substantial differences in the level of happiness, health, cultural context, wealth, as well as health and social care systems across countries [17], investigating the performance of the CarerQol instrument in a sample of informal caregivers from another European region can add valuable information about the cross-cultural validity of this instrument. In addition, previous validations were conducted in distinct samples of caregivers that for instance cared for adult care recipients with a chronic disease or health problems due to ageing [7, 12, 18–20], for caregivers of patients in palliative care [16] or for children with special health needs [21, 22]. However, there is less experience with the CarerQol when used in online samples of the general population [19, 20] although there is an increasing trend in using online surveys. The administration mode of the CarerQol may influence response patterns [20], therefore we aimed to test the online version in the Hungarian context.

Most CarerQol validation studies included the EQ-5D-3L, a generic health status measure that involves a 3-level response scale to report problems in five health domains [23]. The recently developed EQ-5D-5L version (with a 5-level response scale) has been proved to have better measurement properties than the EQ-5D-3L in samples from the general population [24–27]. To our knowledge, the EQ-5D-5L was used in combination with the CarerQol in two studies [13, 28], but these did not focus on the validity of the CarerQol. Hence, investigating the associations between the CarerQol and EQ-5D-5L can provide valuable information about the validity of the CarerQol instrument and for estimating caregiver utilities from care recipient health data when caregiver data are not available. Utilities represent societal preferences for or the value people attach to different health or caregiving states. Using available tariffs, scores on the EQ-5D-5L and the CarerQol can be turned into utility values that can be included in the effect-side of cost–utility analyses of interventions.

The aims of our study were, therefore, to develop the Hungarian version of the CarerQol instrument and assess its construct validity alongside the EQ-5D-5L questionnaire in an online sample of informal caregivers in the general population.

## Methods

### Data collection

The data presented here were part of a larger three-country survey (Hungary, Poland and Slovenia), details have been published elsewhere [29]. The survey consisted of two main modules. The objective of the first module was to obtain country-specific population tariffs for the CarerQol-7D instrument [30]. The second module (reported in this paper) focused on the subsample of current informal caregivers and their care-related quality of life. A cross-sectional online survey was conducted in November 2018. The web-based questionnaire was distributed by a professional survey company, Big Data Scientist Kft. Respondents were recruited from a large online panel of this survey company. Quotas were applied to ensure the representativeness of the sample (*N* = 1000) for the general adult population by age, gender, educational level and residency.

### The questionnaire and selection of informal caregivers

Data were collected on the social-demographic characteristics of respondents (such as age, gender, education, marital status and current employment status), the household of the respondent (size, monthly net income), the place of residence (settlement type, geographical region) and the health status of the respondent (self-perceived health: excellent/very good/good/fair/poor; and the EQ-5D-5L). To select informal caregivers, first a detailed definition of informal care was provided. Then, respondents were asked about their experience with informal care, either as a recipient or a provider, details have been published elsewhere [29] (Online Resource 1). We followed the same method to select informal caregivers for the analyses [29]: only those respondents were included who selected the “I have been providing care or support to a family member or friend for a long period of time” response in the survey. We did not apply any restrictions regarding the characteristics of the care recipient.

Informal caregivers were asked to complete the CarerQol-7D instrument, and to provide further details on their caregiving situation, including the nature of health problems of the care recipient (mainly mental, mainly physical or both), the health status of the care recipient (using the EQ-5D-5L Proxy questionnaire and the health status scale: excellent/very good/good/fair/poor), and the stability of the health status of the care recipient (no improvement is expected in the future but worsening may occur; improvement is expected in the future, do not know), the relationship to the care recipient (partner, parent, mother/father-in-law, child, brother/sister, grandparent, uncle/aunt, other family member, neighbour, friend), the time allocated to the caregiving tasks (hours per week), for how long the respondent had been providing care to the care recipient (duration of caregiving) and if the care recipient lived in the same household (and if not, the travel time to get there). We also asked respondents to evaluate their overall caregiving experience, i.e. whether caregiving affected the respondent’s life negatively, positively or neither negatively nor positively. The survey questionnaire was pilot tested on five respondents in Hungary.

### The instruments

The CarerQol instrument [7] consists of two parts. The first part, the CarerQol-7D descriptive system, includes two positive (fulfilment and support) and five negative (relational problems, mental health problems, problems combining daily activities with care, financial problems and physical health problems) caregiving domains, each with three response levels (none, some, a lot). The second part, the CarerQol-VAS, measures current happiness of caregivers on a horizontal visual analogue scale (VAS) ranging from 0 (completely unhappy) to 10 (completely happy). The answers on the CarerQol-7D descriptive system jointly describe a caregiving situation. For each caregiving situation defined by the descriptive system, a preference-based index score (or utility score) between 0 (representing the worst possible caregiving situation) and 100 (representing the best possible caregiving situation) can be computed. Tariff sets derived from representative samples of the general public are available for several countries [31, 32]. Since Hungarian tariffs are not yet available, CarerQol-7D scores were calculated using the Dutch tariffs [32].

The EQ-5D-5L is a generic health status measure [24]. The questionnaire consists of two parts. First, a descriptive system covering five domains of health: mobility, self-care, usual activities, pain/discomfort and anxiety/depression. Respondents are asked to indicate the level of problems per domain (no problems—1, slight problems—2, moderate problems—3, severe problems—4 and extreme problems/unable to—5) that best describes their current health status. Utility tariffs have been established for the health states described by the instrument for several countries, reflecting the preferences of the general public for the different health states. Due to a lack of country-specific tariffs for Hungary [33], we used the tariffs for The Netherlands in our study (value range: − 0.446 reflecting extreme problems on all domains; 1.0 reflecting no problems in any of the five domains) [34]. Second, the instrument contains a vertical visual analogue scale (EQ VAS), with anchors referring to the worst (0) and best (100) imaginable health state. Respondents are asked to indicate their current health on this scale. We applied the proxy version (EQ-5D-5L Digital Proxy1 Web) to assess the care recipients’ health status, based on caregivers’ responses, as we did not have direct access to care recipients.

### The development of the Hungarian version of the CarerQol-7D questionnaire

A Hungarian language version of the CarerQol-7D was developed by independent forward and backward translations and pilot tests. Two independent forward translations from English to Hungarian were performed. The native in-country principal investigator reviewed the forward translations, discussed the differences with the team and the developers of the original CarerQol to produce a reconciled Hungarian version that most accurately represents the concepts within the source version. This version was back translated into English by two independent professional translators (blinded to the original English wording). Translations were checked for accuracy and were compared to the original English language questionnaire, and minor changes of the Hungarian wording were made (e.g. reconciled forward translation of ‘fulfilment’ was ‘megelégedést okoz’; back translations were ‘fulfilment’ and ‘satisfaction’; the final Hungarian version was ‘elégedettséggel tölt el’). After this, cognitive debriefing was conducted in face-to-face interviews with five respondents, before finalising the Hungarian language version.

### Ethics

Ethical approval was obtained from the Hungarian Medical Research Council (Nr. 35286-2/2018/EKU). Respondents were informed that participation in the survey was completely voluntary, that their data would remain anonymous and would not be linked to personal information, such as their name or address, and that their data would be used solely for scientific purposes. Respondents needed to provide their informed consent before the start of the survey.

### Analysis

The psychometric performance of the CarerQol-7D was evaluated in relation to the health status of both the informal caregiver and the care recipient, as measured with the EQ-5D-5L [24], and caregiving situation characteristics. The construct validation included clinical, convergent and discriminant validity tests. Subgroup comparisons were carried out by non-parametric tests (Mann–Whitney *U* and Kruskal–Wallis tests). Spearman correlation coefficients were calculated between continuous variables. In all types of analysis, a 5% significance level was applied. The strength of correlation coefficients (*r*) was assessed as follows [35] *r* < 0.1: trivial; 0.1 ≤ *r* < 0.3: small; 0.3 ≤ *r* < 0.5: moderate; 0.5 ≤ *r* < 0.7: high; 0.7 ≤ *r* < 0.9: very high; and 0.9 ≤ *r*: nearly perfect. All statistical analyses were carried out in SPSS Statistics 25.

#### Clinical validity

We assessed whether CarerQol-7D and CarerQol-VAS scores were responsive to different caregiving situations. For categorical variables, we analysed whether CarerQol-7D and CarerQol-VAS scores differed significantly by relevant characteristics of the caregiving situation: the nature of health problems of the care recipient (mainly mental, mainly physical, or both); the health status of the care recipient; stability of the health status of the care recipient; relationship to the care recipient; duration of caregiving (less than 6 months; 7–12 months; more than 1 year); and, finally, if the care recipient lived in the same household as the respondent.

We calculated correlations between CarerQol-7D scores, CarerQol-VAS scores and the health status of the care recipient (EQ-5D-5L index score and EQ VAS Proxy versions), and the weekly time allocated to the caregiving tasks. We also explored whether individual EQ-5D-5L domain scores were associated with the CarerQol-7D and CarerQol-VAS scores.

Based on the results of previous validation studies, we expected significant association of CarerQol-7D and CarerQol-VAS scores with the care recipients’ health status, with lower scores associated with worse health status [16, 19] and with mental health problems [7]. Moreover, we expected the type of relationship between the caregiver and care recipient to be associated with CarerQoL-7D and CarerQol-VAS scores [7, 12, 19], lower scores with longer duration of caregiving [12, 16], and lower scores in case the caregiver and care recipient live in the same household [7], ceteris paribus.

#### Discriminant validity

We examined whether scores for the CarerQol-VAS, EQ-5D-5L index and EQ VAS of care recipients on the three different levels of the seven CarerQol-7D domains (no/some/a lot of problems) differed significantly. Based on previous studies, we expect significantly higher CarerQol-VAS score when the caregiver reports no problems with the caregiving domains, or more fulfilment and support [19].

#### Convergent validity

First, we expected positive associations of CarerQol-VAS scores with the two positive CarerQol-7D domains (fulfilment and support) and negative associations with the five negative CarerQol-7D domains [19]. Convergent validity of the CarerQol-7D was also evaluated by studying the associations between CarerQol-7D scores and CarerQol-VAS scores and EQ-5D-5L and EQ VAS scores of caregivers. Second, we examined the correlations between the CarerQol-7D and EQ-5D-5L domains. We expected a high correlation between the “Mental health problems” domain of the CarerQol-7D questionnaire and the “Anxiety/depression” domain of the EQ-5D-5L questionnaire. Also, we expected a significant association between the “Physical health problems” domain of the CarerQol-7D questionnaire and the “Mobility” and “pain/discomfort” domains of the EQ-5D-5L questionnaire. Thirdly, we compared CarerQol-7D and CarerQol-VAS scores to the overall caregiving experience of caregivers. We expected higher scores to be associated with more positive care experiences.

## Results

Among the 1,000 respondents (female 51.2%; mean age 53.2, SD 15.2), 149 informal caregivers were identified [29]. The average age of caregivers was significantly higher than that of the general population in our sample (56.1 years, SD 14.2 vs 52.6, SD 15.2, respectively), while the share of women (58.4% vs 49.9%, *p* = 0.057) was not significantly higher. The average household size of caregivers was 2.4 persons (SD 1.3), and their per capita household income was 403 EUR (SD 245) and it did not differ significantly (*p* = 0.305) from that of the non-caregivers’ subgroup (conversion €1 = 322 HUF).

**Fig. 1:**
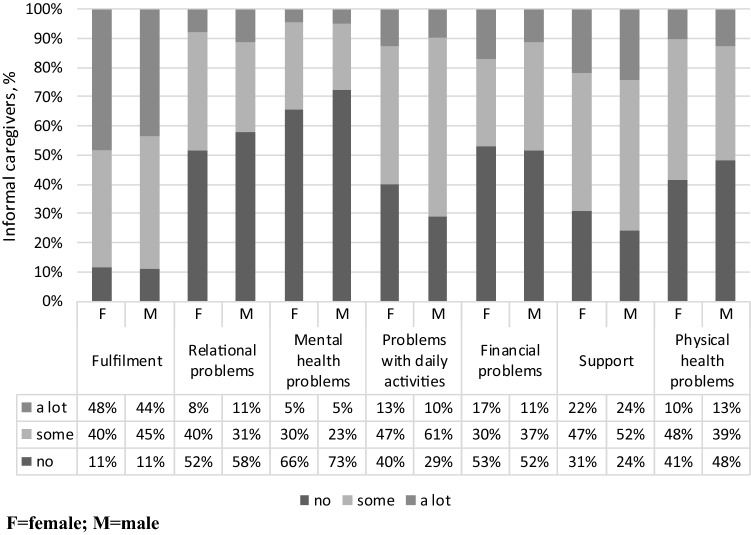
Distribution of CarerQol-7D domain scores reported by informal caregivers. *F* female, *M* male

The average Carerqol-7D and CarerQol-VAS scores of caregivers were 76.0 (SD 16.2) and 6.8 (SD 2.3), respectively. CarerQol-7D and CarerQol-VAS scores did not differ significantly between sociodemographic subgroups but did differ with self-perceived health status (Table 1). The distribution of informal caregivers’ responses across the seven domains of the CarerQol-7D is presented in Fig. 1. The EQ-5D-5L and the EQ VAS scores of caregivers were 0.830 (SD 0.202) and 75.0 (SD 18.8), respectively, while among the care recipients these scores were 0.466 (SD 0.286) and 47.0 (SD 22.7), respectively. The distribution of health problems across the EQ-5D-5L domains for caregivers and care recipients are presented in a supplementary file (Online Resource 2).

**Table 1:** Sample characteristics, and CarerQol-7D and CarerQol-VAS scores by sociodemographic characteristics of caregivers

	*N*	%	CarerQol-7D score Mean (SD)	CarerQol-VAS score Mean (SD)
Total	149	100		
Gender			*χ*^2^ = 0.142 *p* = 0.706	*χ*^2^ = 0.000 *p* = 0.992
Women	87	58.4	75.6 (16.1)	6.8 (2.4)
Men	62	41.6	76.4 (16.5)	6.8 (2.1)
Age			*χ*^2^ = 8.554 *p* = 0.128	*χ*^2^ = 2.749 *p* = 0.739
18–24	3	2.0	93.6 (8.6)	7.3 (2.5)
25–34	11	7.4	72.9 (22.0)	6.6 (2.5)
35–44	20	13.4	70.0 (21.2)	6.3 (3.0)
45–54	27	18.1	78.2 (14.3)	7.1 (2.6)
55–64	38	25.5	74.4 (14.3)	6.7 (2.0)
65+	50	33.6	76.0 (16.2)	7.0 (2.0)
Education			*χ*^2^ = 0.986 *p* = 0.611	*χ*^2^ = 0.421 *p* = 0.810
Primary	28	18.8	77.7 (17.4)	6.6 (2.6)
Secondary	55	36.9	73.8 (18.4)	6.8 (2.2)
Tertiary	66	44.3	77.0 (13.5)	6.9 (2.2)
Employment			*χ*^2^ = 5.049 *p* = 0.282	*χ*^2^ = 0.944 *p* = 0.918
Employed full time/self-employed	59	39.6	77.0 (16.8)	6.8 (2.5)
Working part time	6	4.0	70.7 (21.9)	7.2 (1.8)
Pensioner	64	43.0	76.1 (14.2)	6.9 (2.0)
Disability pensioner	5	3.4	65.7 (7.6)	6.0 (2.4)
Other	15	10.1	76.9 (21.2)	6.7 (2.8)
Settlement type			*χ*^2^ = 3.398 *p* = 0.183	*χ*^2^ = 0.594 *p* = 0.743
Capital	32	21.5	73.4 (17.0)	7.0 (2.3)
Town	85	57.0	78.4 (14.4)	6.8 (2.2)
Village	32	21.5	71.9 (18.9)	6.8 (2.4)
Married/having a partner			*χ*^2^ = 0.032 *p* = 0.858	*χ*^2^ = 3.004 *p* = 0.083
Yes	101	67.8	76.4 (14.7)	7.0 (2.3)
No	48	32.2	75.1 (19.0)	6.4 (2.3)
Self-reported health			*χ*^2^ = 23.617 *p* = 0.000	*χ*^2^ = 22.015 *p* = 0.000
Excellent	9	6.0	79.3 (21.5)	8.0 (2.1)
Very good	28	18.8	81.3 (14.6)	7.7 (1.8)
Good	67	45.0	79.1 (13.9)	7.0 (2.1)
Fair	37	24.8	68.9 (16.5)	6.0 (2.3)
Poor	8	5.4	59.6 (16.2)	4.1 (2.8)
Household size (number of persons)			*χ*^2^ = 5.130 *p* = 0.274	*χ*^2^ = 2.558 *p* = 0.634
1	31	20.8	76.3 (18.9)	6.8 (1.9)
2	58	38.9	75.4 (16.5)	6.8 (2.4)
3	40	26.8	75.4 (14.2)	6.6 (2.1)
4	12	8.1	72.5 (15.5)	7.2 (2.4)
5–7	8	5.3	86.4 (11.4)	7.1 (3.6)
Income category*			*χ*^2^ = 0.991 *p* = 0.803	*χ*^2^ = 3.231 *p* = 0.357
0–621 EUR	45	30.2	74.0 (17.4)	6.7 (2.3)
622–932 EUR	44	29.5	76.1 (15.1)	6.6 (2.3)
Above 932 EUR	45	30.2	77.5 (16.6)	7.2 (2.2)
I do not want to answer	15	10.1	76.9 (15.0)	6.3 (2.7)

Differences of means were tested by Kruskal–Wallis test and Mann–Whitney *U* test

*Conversion: 1 EUR = 322 HUF (the exchange rate in November, 2018)

### Clinical validity

Table 2 presents the CarerQol-7D and CarerQol-VAS scores by characteristics of the caregiving situation. CarerQol-7D scores were significantly associated (*p* < 0.05) with the nature of the care recipients’ health problems, the lowest scores were found for caregivers of persons with mental health problems only (69.4). The difference between subgroups ‘mainly mental health problems’ and ‘both physical and mental health problems’ was not statistically significant. CarerQol-VAS scores also did not differ significantly across the three categories.

**Table 2:** CarerQol-7D and CarerQol-VAS scores by characteristics of caregiving situations

	No.	%	CarerQol-7D index score, caregivers Mean (SD)	CarerQol-VAS (happiness) score, caregivers Mean (SD)
Nature of health problem (care recipient)			*χ*^2^ = 7.919 *p* = 0.019	*χ*^2^ = 1.152 *p* = 0.562
Mostly physical problems	73	49.0	79.6 (14.4)	6.9 (2.1)
Mostly mental problems	26	17.4	69.4 (20.7)	7 (2.5)
Both	50	33.6	74 (14.8)	6.5 (2.4)
Health status of the care recipient			*χ*^2^ = 9.810 *p* = 0.020	*χ*^2^ = 2.626 *p* = 0.453
Excellent/very good	8	5.4	75.0 (19.9)	7.5 (1.6)
Good	25	16.8	74.8 (16.6)	6.6 (2.1)
Fair	90	60.4	78.5 (15.4)	7 (2.2)
Poor	26	17.4	68.5 (15.8)	6.1 (2.9)
Future improvements in care recipients’ health status			*χ*^2^ = 8.059 *p* = 0.045	*χ*^2^ = 5.398 *p* = 0.145
No improvement is expected in the future (worsening may occur)	108	72.5	76.1 (15.9)	6.8 (2.3)
It is expected to improve in the future	26	17.4	80 (13.8)	7.1 (2.2)
I don’t know	14	9.4	65.8 (19.3)	5.9 (2.3)
I don’t want to answer	1	0.7	93.7	10.0
Care provided for			*χ*^2^ = 4.670 *p* = 0.097	*χ*^2^ = 1.639 *p* = 0.441
≤ 6 months	12	8.1	66.8 (18.8)	6.5 (2.4)
7–12 months	18	12.1	78.8 (14.3)	6.2 (2.6)
More than 1 year	117	78.5	76.6 (15.9)	6.9 (2.2)
Relation of care recipient to the caregiver^a^			*χ*^2^ = 9.716 *p* = 0.084	*χ*^2^ = 3.563 *p* = 0.614
Partner	19	12.8	71.3 (14.6)	6.4 (2.2)
Parent	53	35.6	76.3 (14)	6.5 (2.5)
In-law	18	12.1	78.2 (11.8)	7.7 (1.4)
Child	12	8.1	73.6 (14)	6.8 (1.7)
Other family member	29	19.5	73.5 (21.4)	7.1 (2.1)
Neighbour/friend	18	12.1	83.2 (18.2)	6.7 (2.8)
Living in the same household			*χ*^2^ = 5.948 *p* = 0.015	*χ*^2^ = 3.585 *p* = 0.058
Yes	51	34.2	72.3 (15.5)	6.4 (2.2)
No	98	65.8	77.9 (16.2)	7 (2.3)
Overall care experience			*χ*^2^ = 24.867 *p* = 0.000	*χ*^2^ = 17.213 *p* = 0.000
Rather negative	43	28.9	65.6 (16.3)	5.4 (2.6)
Neither negative, nor positive	69	46.3	80.2 (11.9)	7.2 (2)
Rather positive	37	24.8	80.1 (17.8)	7.6 (1.7)

Differences of means were tested by Kruskal–Wallis test

^a^The difference was statistically significant between ‘any family member’ (joint analysis of ‘partner’, ‘parent’, ‘parent in-law’, ‘child’ categories) and ‘friends or neighbours’ subgroups (*p* = 0.009)

CarerQol-7D scores were significantly associated with the health status proxy for the care recipient. When comparing subgroups, only CarerQol-7D scores for caregivers of persons with ‘Poor’ health status differed significantly from the other groups. CarerQol-VAS score was not significantly associated with the health status proxy scores of the care recipient.

We found the highest average CarerQol-7D score for caregivers of friends or neighbours (83.2, SD 18.2), and the lowest average score for caregivers of their partners (71.3, SD 14.6) (Table 2). CarerQol-7D scores were significantly higher in case the care recipient’s health status was expected to improve in the future, significantly lower (indicating higher burden) if the care was provided for less than 6 months (compared to > 6 months) and when the caregiver and care recipient lived in the same household (compared to those who do not live in the same household). Caregivers’ happiness (CarerQol-VAS scores) did not show significant differences between these categories.

Regarding correlations, we found small but significant correlations between CarerQol-7D scores, CarerQol-VAS scores and caregiving time (*r* = − 0.257 and − 0.212). The correlation between the CarerQol-7D scores and EQ-5D-5L scores (proxy) of the care recipients was small (*r* = 0.247) (Table 3). No significant correlations were found between the CarerQol-7D scores and EQ VAS scores (proxy) of the care recipients (*r* = 0.144), or between caregivers’ happiness (CarerQol-VAS scores) and care recipients’ health status (EQ-5D-5L score proxy: *r* = 0.054; EQ VAS proxy: *r* = 0.076). We found small but significant correlations between the CarerQol-7D scores and three (‘Usual activities’, ‘Pain/discomfort’, ‘Anxiety/depression’) out of the five domains of the care recipients’ EQ-5D-5L (proxy) questionnaire. We found no significant correlations between caregivers’ happiness (CarerQol-VAS) and care recipients’ EQ-5D-5L (proxy) domains. There were some small but significant correlations between some of the care recipients’ EQ-5D-5L domains and CarerQol-7D domains (Table 3).

**Table 3:** Spearman correlations of CarerQol-7D and CarerQol-VAS scores with EQ-5D-5L domains, EQ-5D-5L scores and EQ VAS scores of caregivers and care recipients

	CarerQol-7D Fulfilment	CarerQol-7D Relational problems	CarerQol-7D Mental health problems	CarerQol-7D Problems with daily activity	CarerQol-7D Financial problems	CarerQol-7D Support	CarerQol-7D Physical health problems	CarerQol-7D score	CarerQol-VAS score
Caregiver									
EQ-5D-5L Mobility	− 0.105 *p* = 0.203	0.088 *p* = 0.289	0.098 *p* = 0.234	0.139 *p* = 0.092	0.145 *p* = 0.078	0.012 *p* = 0.884	0.526** *p* = 0.000	− 0.364** *p* = 0.000	− 0.140* *p* = 0.089
EQ-5D-5L Self-care	− 0.066 *p* = 0.422	0.098 *p* = 0.235	0.092 *p* = 0.266	0.132 *p* = 0.107	0.143 *p* = 0.082	0.028 *p* = 0.731	0.222** *p* = 0.006	− 0.197* *p* = 0.016	− 0.169** *p* = 0.039
EQ-5D-5L Usual activities	− 0.028 *p* = 0.732	0.092 *p* = 0.265	0.154 *p* = 0.06	0.206* *p* = 0.012	0.120 *p* = 0.146	0.067 *p* = 0.420	0.516** *p* = 0.000	− 0.335** *p* = 0.000	− 0.222** *p* = 0.007
EQ-5D-5L Pain/discomfort	0.003 *p* = 0.972	0.031 *p* = 0.709	0.228** *p* = 0.005	0.085 *p* = 0.305	0.228** *p* = 0.005	0.045 *p* = 0.584	0.544** *p* = 0.000	− 0.353** *p* = 0.000	− 0.278** *p* = 0.001
EQ-5D-5L Anxiety/depression	− 0.019 *p* = 0.823	0.225** *p* = 0.006	0.548** *p* = 0.000	− 0.029 *p* = 0.725	0.107 *p* = 0.195	0.074 *p* = 0.372	0.310** *p* = 0.000	− 0.343** *p* = 0.000	− 0.344** *p* = .000
EQ-5D-5L index score	0.042 *p* = 0.615	− 0.137 *p* = 0.096	− 0.352** *p* = 0.000	− 0.096 *p* = 0.244	− 0.210* *p* = 0.010	− 0.057 *p* = 0.493	− 0.632** *p* = 0.000	0.453** *p* = 0.000	0.326** *p* = 0.000
EQ VAS score	0.083 *p* = 0.311	− 0.056 *p* = 0.5	− 0.158 *p* = 0.054	− 0.167* *p* = 0.042	− 0.188* *p* = 0.022	0.028 *p* = 0.739	− 0.504** *p* = 0.000	0.387** *p* = 0.000	0.242** *p* = 0.003
Care recipient (EQ-5D-5L Proxy)									
EQ-5D-5L Mobility	− 0.040 *p* = 0.632	− 0.052 *p* = 0.526	− 0.076 *p* = 0.358	0.122 *p* = 0.140	− 0.079 *p* = 0.336	0.046 *p* = 0.574	0.014 *p* = 0.864	− 0.001 *p* = 0.990	− 0.008 *p* = 0.925
EQ-5D-5L Self-care	− 0.020 *p* = 0.805	0.071 *p* = 0.388	0.006 *p* = 0.944	0.169* *p* = 0.039	0.094 *p* = 0.254	0.162* *p* = 0.048	0.082 *p* = 0.322	− 0.141 *p* = 0.087	− 0.007 *p* = 0.931
EQ-5D-5L Usual activities	− 0.010 *p* = 0.901	0.105 *p* = 0.202	0.013 *p* = 0.879	0.207* *p* = 0.011	0.147 *p* = 0.074	0.057 *p* = 0.488	0.112 *p* = 0.173	− 0.209* *p* = 0.010	0.014 *p* = 0.868
EQ-5D-5L Pain/discomfort	− 0.172* *p* = 0.036	0.170* *p* = 0.039	0.070 *p* = 0.394	0.053 *p* = 0.522	0.166* *p* = 0.043	− 0.054 *p* = 0.512	0.120 *p* = 0.146	− 0.222** *p* = 0.007	− 0.111 *p* = 0.176
EQ-5D-5L Anxiety/depression	0.003 *p* = 0.968	0.228** *p* = 0.005	0.239** *p* = 0.003	0.173* *p* = 0.035	0.262** *p* = 0.001	0.113 *p* = 0.170	0.169* *p* = 0.039	− .0247** *p* = 0.002	− 0.089 *p* = 0.279
EQ-5D-5L index score	0.100 *p* = 0.225	− 0.199* *p* = 0.015	− 0.136 *p* = 0.099	− 0.196* *p* = 0.016	− 0.186* *p* = 0.023	− 0.105 *p* = 0.203	− 0.121 *p* = 0.142	0.247** *p* = 0.002	0.054 *p* = 0.516
EQ VAS score	0.001 *p* = 0.995	− 0.001 *p* = 0.990	0.058 *p* = 0.480	− 0.092 *p* = 0.267	− 0.110 *p* = 0.182	− 0.047 *p* = 0.570	− 0.134 *p* = 0.102	0.144 *p* = 0.080	0.076 *p* = 0.357

*Correlation is significant at the 0.05 level

**Correlation is significant at the 0.01 level

### Discriminant validity

Table 4 presents the CarerQol scores of respondents for the different levels per CarerQol-7D domains (e.g. no/some/a lot of problems). CarerQol-VAS scores were higher among caregivers that experienced fulfilment and received support and when problems were absent. However, differences between CarerQol-VAS scores were not significant for the domains ‘Problems with daily activities’, ‘Financial problems’ and ‘Support’. Both EQ-5D-5L scores and EQ VAS were significantly higher for caregivers with no mental health problems and no physical health problems.

**Table 4:** CarerQol and EQ-5D-5L scores of respondents on each level of the CarerQol-7D domains

CarerQol-7D domain	*N* (%)	CarerQol-VAS (happiness) score Mean (SD)	EQ-5D-5L score Mean (SD)	EQ VAS score Mean (SD)
Fulfilment		*χ*^2^ = 9.766 *p* = 0.008	*χ*^2^ = 0.258 *p* = 0.879	*χ*^2^ = 2.658 *p* = 0.265
None	17 (11.4%)	5.7 (3.1)	0.777 (0.291)	75.1 (22.8)
Some	63 (42.3%)	6.4 (2.1)	0.835 (0.189)	73.5 (16.8)
A lot	69 (46.3%)	7.4 (2.1)	0.839 (0.188)	76.4 (19.7)
Relational problems		*χ*^2^ = 6.675 *p* = 0.036	*χ*^2^ = 4.034 *p* = 0.133	*χ*^2^ = 0.844 *p* = 0.656
None	81 (54.4%)	7.2 (2.2)	0.850 (0.177)	75.4 (19.4)
Some	54 (36.2%)	6.5 (2.4)	0.820 (0.227)	75.7 (16.9)
A lot	14 (9.4%)	5.9 (1.9)	0.750 (0.223)	70.8 (23.2)
Mental health problems		*χ*^2^ = 12.650 *p* = 0.002	*χ*^2^ = 19.172 *p* = 0.000	*χ*^2^ = 8.846 *p* = 0.012
None	102 (68.5%)	7.3 (2)	0.870 (0.168)	76 (19.1)
Some	40 (26.8%)	5.8 (2.6)	0.792 (0.171)	76.8 (14.4)
A lot	7 (4.7%)	5.3 (2.9)	0.475 (0.390)	51.6 (23.9)
Problems with daily activities		*χ*^2^ = 3.668 *p* = 0.160	*χ*^2^ = 1.399 *p* = 0.497	*χ*^2^ = 4.889 *p* = 0.087
None	53 (35.6%)	7.3 (2.2)	0.869 (0.127)	78.5 (17.9)
Some	79 (53.0%)	6.7 (2.1)	0.817 (0.221)	72.9 (19.4)
A lot	17 (11.4%)	5.8 (3)	0.768 (0.273)	74 (18.6)
Financial problems		*χ*^2^ = 4.015 *p* = 0.134	*χ*^2^ = 7.056 *p* = 0.029	*χ*^2^ = 5.257 *p* = 0.072
None	78 (52.3%)	7.1 (2.3)	0.857 (0.189)	77.6 (18.7)
Some	49 (32.9%)	6.5 (2.2)	0.830 (0.183)	73.4 (16.8)
A lot	22 (14.8%)	6.5 (2.4)	0.736 (0.257)	69.3 (22.3)
Support		*χ*^2^ = 4.911 *p* = 0.086	*χ*^2^ = 0.557 *p* = 0.757	*χ*^2^ = 0.163 *p* = 0.922
None	42 (28.2%)	6.3 (2.8)	0.855 (0.162)	73.5 (21.1)
Some	73 (49.0%)	6.7 (2.1)	0.813 (0.235)	75.6 (16.8)
A lot	34 (22.8%)	7.6 (1.8)	0.837 (0.166)	75.7 (20.3)
Physical health problems		*χ*^2^ = 7.069 *p* = 0.029	*χ*^2^ = 60.582 *p* = 0.000	*χ*^2^ = 42.203 *p* = 0.000
None	66 (44.3%)	7.3 (2.2)	0.917 (0.165)	82.1 (16.5)
Some	66 (44.3%)	6.5 (2.2)	0.820 (0.130)	75 (14.2)
A lot	17 (11.4%)	5.9 (2.7)	0.530 (0.262)	47.5 (18.3)

Differences in means were tested by Kruskal–Wallis test

### Convergent validity

CarerQol-VAS scores were positively associated with the positive domains of the CarerQol-7D, and negatively with the negative domains of CarerQol-7D. Correlation was significant between CarerQol-7D and CarerQol-VAS scores (*r* = 0.363). As shown in Table 3, the correlation between CarerQol-7D scores with EQ-5D-5L scores and EQ VAS scores of caregivers were significant and moderate (*r* = 0.453 and 0.387, respectively). The strength of correlation between CarerQol-VAS and EQ-5D-5L scores was significant and moderate (*r* = 0.453) and between CarerQol-VAS and EQ VAS scores was significant but small (*r* = 0.242). Both CarerQol-7D scores and CarerQol-VAS scores significantly correlated with all EQ-5D-5L domain scores (small to moderate correlations).

Regarding the correlations between domains of the CarerQol-7D and EQ-5D-5L instruments assessing the caregivers’ status, the highest correlations were found between the ‘mental health problems’ and the ‘anxiety and depression’, and the ‘physical health problems’ and ‘pain/discomfort’ (and ‘mobility’) domains, respectively (Table 3).

As Table 2 shows, caregivers’ CarerQol-7D and CarerQol-VAS scores differed significantly by the overall care experience. However, both for CarerQol-7D and CarerQol-VAS scores, this difference was not significant between the ‘neither negative nor positive’ and ‘rather positive’ categories. Similar relationships were found between overall care experience and the EQ-5D-5L scores of the care recipients.

## Discussion

We developed the Hungarian language version of the CarerQol instrument and assessed its psychometric performance in a sample of informal caregivers in the general population. We evaluated the Hungarian version of the CarerQol instrument in relation to the health status of caregivers and care recipients, measured by the EQ-5D-5L questionnaire, and several characteristics of the caregiving situation. The analysis involved clinical, convergent and discriminant validity tests. This is the first validation study of this instrument in the Central and Eastern European region.

Regarding clinical validity, our findings are in line with results of previous validation studies [7, 12, 19]. First, we observed a negative association of the CarerQol-7D scores with the care recipients’ health status (measured by the EQ-5D-5L proxy score). Secondly, a negative association was observed with the duration of caregiving. Thirdly, similarly to findings by Brouwer et al. [7], lower CarerQol-7D scores were observed when the caregiver and the care recipient lived in the same household (compared to when this was not the case), and CarerQol-7D scores were also lower when the care receiver mainly suffered from mental health problems. Fourthly, similarly to Brouwer et al. [7] and Hoefman et al. [19], we also found associations between the CarerQol-7D and the type of relationship between the caregiver and care recipient. In our case, caring for a neighbour or friend was associated with higher care-related quality of life, while caring for a partner was associated with relatively lower scores. We can hypothesise that care recipients who are neighbours or friends may have additional helpers (family members, paid carers) as well. Probably, a spouse or partner may experience shared financial concerns or problems with the care recipient and may experience more care-related burden. Care time may also have effect on this relationship. These issues deserve further joint investigation in future studies involving larger samples. Some of the previous studies also reported relationship between CarerQol-VAS score and income [7, 19] or employment status [19]. However, we did not find a significant association between these variables in our sample. Nevertheless, the similarities to previous results strongly support the clinical validity of the Hungarian version of the instrument.

One strength of our study is that we included the overall CarerQol-7D index scores which have been applied before only in two validity tests [16, 20]. The CarerQol-7D index score performed better in distinguishing the burden of caregiving, than the general happiness measure, the CarerQol-VAS. Moreover, we focused on the convergent validity of the CarerQol-7D in relation to the EQ-5D-5L instrument, which relation has not been extensively studied before. With the EQ-5D-5L, we measured health-related quality of life of the caregivers and assessed if this was associated with their care-related quality of life. The significant moderate correlation between CarerQol-7D and EQ-5D-5L index score highlights a certain overlap between the two measures. Hence, when evaluating interventions for caregivers, there is a risk of double counting effects when both measures are used. On the other hand, the moderate correlation between CarerQol-7D and EQ-5D-5L proxy scores for care recipients indicates that estimation of CarerQol scores from EQ-5D-5L scores of care recipients should be done only with caution [36]. Alternatively, caregiving time could be estimated, and informal care included on the cost-side of an economic evaluation. Our EQ-5D-5L results moreover suggest that the prevalence of health problems is rather high among caregivers, especially in the ‘Pain/discomfort’ domain. This implies that a substantial proportion of caregivers had to cope with their own health problems while caring for someone else in their social network. Furthermore, we observed that in certain situations it was not the health status of the care recipient but other care-related factors (such as living arrangements, the relation to the care recipient, duration of care and expectations regarding future health improvements) that were associated with caregivers’ quality of life. These findings indicate that the context of caregiving should be considered as seriously as the health status of the care recipient when developing health and social care interventions that aim to support informal caregivers.

### Limitations

Some limitations of our study need to be noted. The overall sample was representative in terms of main sociodemographic characteristics, but only up to the age of 65, thus only few elderly caregivers were included in our study. Hence, spousal caregiving, which is frequent in later life, as well as aspects of informal caregiving that are more typical in the elderly (e.g. the impact of their own disabilities and limitations, fear from the future) were probably only partly explored. Given that the survey was administered among an online panel, representativeness for the entire population is limited (selection bias). Informal caregivers who do not use internet could not be reached (coverage bias) and relatively few heavily burdened caregivers were involved (non-response bias). In our study, we did not consider whether the included caregivers were providing care for one or more individuals, or whether they were the primary caregivers of the care recipient or not. Even though our total sample of 1000 was sizeable, the sample size of the caregivers’ subgroup was relatively small (*N* = 149), which limits the statistical power of our analyses. Furthermore, health status of the care recipients was not assessed directly but by proxy measures. Finally, we could not apply Hungarian tariffs for the CarerQol and the EQ-5D-5L instruments, as these were not yet available.

## Conclusions

The Hungarian language version of the CarerQol appears to be a valid instrument to assess the care-related quality of life of informal caregivers in Hungary, when applied in an online sample of informal caregivers in the general population. Strong similarities with validation studies from The Netherlands support the cross-cultural validity of the CarerQol. Further prospective studies are encouraged to replicate this finding in other groups of caregivers, including different (disease-specific) subgroups of caregivers and different caregiving situations. Moreover, future studies could assess the test–retest reliability and responsiveness of the instrument to changes. Our findings suggest that caregivers’ care-related quality of life and happiness are not perfectly correlated with the health status of the care recipient. Caregivers’ own general health and the context of caregiving, including personal factors and external conditions, seem to have more effect on care-related quality of life. This is relevant to consider when developing health and social care policy strategies that aim to support informal caregivers.

## Electronic supplementary material

Below is the link to the electronic supplementary material.

Supplementary file1 (DOCX 17 kb)

Supplementary file2 (DOCX 36 kb)

## Data Availability

The data that support the findings of this study are available from the corresponding author upon reasonable request.
