# Supplementary material for: Validation of the Hungarian version of the CarerQol instrument in informal caregivers: results from a cross-sectional survey among the general population in Hungary
Source: Qual Life Res. 2020 Oct 10;30(2):629–41. doi: 10.1007/s11136-020-02662-8 (PMC7886830; doi:10.1007/s11136-020-02662-8)
Supplement: Supplementary file 1 — Supplementary file1 (DOCX 17 kb) [file 11136_2020_2662_MOESM1_ESM.docx]

**Title: Validation of the Hungarian version of the CarerQol instrument in informal caregivers: results from a cross-sectional survey among the general population in Hungary**

**Journal: Quality of Life Research**

**Authors: Petra Baji, Werner B.F. Brouwer, Job van Exel, Dominik Golicki, Valentina Prevolnik Rupel, Zsombor Zrubka, László Gulácsi, Valentin Brodszky, Fanni Rencz, Márta Péntek**

**Correspondence: Márta Péntek M.D., Ph.D.; Corvinus University of Budapest;** [**marta.pentek@uni-corvinus.hu**](mailto:marta.pentek@uni-corvinus.hu)**; pentek.marta@uni-obuda.hu**

**Online Resource 1:**

Explanation about informal care provided for the participants in the survey and the question applied to assess their experience with informal care

| **“What is informal care?** |
| --- |
| - Informal care is the care for a family member or friend who needs support due to an illness, disability or infirmity of old age. |
|  |
| - Unpaid carers are often **relatives, friends or acquaintances** of the persons receiving care or support. |
| - Informal carers perform **different type of tasks**, for example: |
| - domestic help, personal care or nursing care; |
| - emotional support, supervision; |
| - accompanying on visits or performing administrative tasks. |
| - Informal carers often provide care for **a long time,** usually for many years. |
| - Caregiving is often **time-consuming.** The time spent on caregiving can range from a couple of hours to more than 40 hours per week. |
| - Informal carers often experience providing care as pleasant and a **natural thing** to do. |
| - However, caregiving can also be **burdensome**.” |

**Experience with informal care**

“Are you familiar with informal carers in your own family or friends?
Choose the answer that is most applicable to you.

- No
- Yes, I have been receiving care or support from a family member or friend for a long period of time
- Yes, I have received care or support from a family member or friend for a long period of time
- Yes, I have been providing care or support to a family member or friend for a long period of time
- Yes, I have provided care or support to a family member or friend for a long period of time during the past year
- Yes, I have provided care or support to a family member or friend for a long period of time in the past
- Yes, I know a person who provides/has provided or receives/has received unpaid care”
