# Supplementary material for: Validation of the Hungarian version of the CarerQol instrument in informal caregivers: results from a cross-sectional survey among the general population in Hungary
Source: Qual Life Res. 2020 Oct 10;30(2):629–41. doi: 10.1007/s11136-020-02662-8 (PMC7886830; doi:10.1007/s11136-020-02662-8)
Supplement: Supplementary file 2 — Supplementary file2 (DOCX 36 kb) [file 11136_2020_2662_MOESM2_ESM.docx]

**Title: Validation of the Hungarian version of the CarerQol instrument in informal caregivers: results from a cross-sectional survey among the general population in Hungary**

**Authors: Petra Baji, Werner B.F. Brouwer, Job van Exel, Dominik Golicki, Valentina Prevolnik Rupel, Zsombor Zrubka, László Gulácsi, Valentin Brodszky, Fanni Rencz, Márta Péntek**

**Correspondence: Márta Péntek M.D., Ph.D.; Corvinus University of Budapest;** [**marta.pentek@uni-corvinus.hu**](mailto:marta.pentek@uni-corvinus.hu)**; pentek.marta@uni-obuda.hu**

**Online Resource 2:**

**Fig. S1 Health problems reported on the EQ-5D-5L descriptive system**
